# EMPLOYMENT OF TRAUMA INFORMED PRINCIPLES IN HEALTH SERVICES RESEARCH WITH OLDER LATINX COMMUNITIES

**DOI:** 10.1093/geroni/igae098.1408

**Published:** 2024-12-31

**Authors:** David Camacho

**Affiliations:** University of Illinois Chicago, Oak Park, Illinois, United States

## Abstract

Engaging older Latinx adults in research is critical to advancing understanding and addressing common challenges in aging. However, older Latinx communities are disproportionately exposed to social, health, and historical challenges that may include potentially traumatic events (e.g. discrimination, violence, grief, etc.) and hinder study participation. There is growing recognition for adoption of Trauma Informed (TI) principles for use in non-trauma specific research. There are limited examples of the implementation of TI principles in gerontological research with Latinx communities. We encourage the adoption of TI principles to facilitate engagement research participation. TI principles may include: 1) building relationships with trusted community agencies and providers; 2) conducting open forums where researchers acknowledge community mistrust and fear, present personal and institutional motivations for the project and discuss benefits for community members; 3)incorporation of study questions that explore Latinx diversity and facilitate discussions about similarities and differences across subgroups; and 4) planning to address structural factors (poverty, violence, migratory status) that influence health and research participation. Second, we draw examples of TI guided practices we employed in two recent studies including the: 1) Dolores and Soledad: a narrative study of older English and Spanish speaking Latinx adults living with chronic pain or loneliness in New York and Los Angeles, and 2) Programa Dolores: An adaptation of the evidence based psychosocial intervention (PATH-Pain) for use with older Latinx adults living with chronic pain in New York. Finally, based on these experiences, we provide recommendations for incorporating TI into future research with older Latinx communities.

